# Plain language summary of a study looking at the effect of empagliflozin treatment in patients with chronic kidney disease

**DOI:** 10.1093/ckj/sfae057

**Published:** 2024-03-07

**Authors:** Alistair J Roddick, Anne Whitehouse, Michaela Petrini, Svenja Seide, Sibylle Hauske

**Affiliations:** Oxford Kidney Unit, Oxford University Hospitals NHS Foundation Trust, Oxford, UK; Communications Team, Nuffield Department of Population Health, University of Oxford, Oxford, UK; Boehringer Ingelheim Pharmaceuticals, Inc., Ridgefield, CT, USA; Boehringer Ingelheim Pharma GmbH & Co. KG, Ingelheim am Rhein, Germany; Boehringer Ingelheim, Ingelheim am Rhein, Germany; Vth Department of Medicine, University Medical Center Mannheim, University of Heidelberg, Mannheim, Germany

## INTRODUCTION

### What is chronic kidney disease (CKD)?

**Figure 1:**
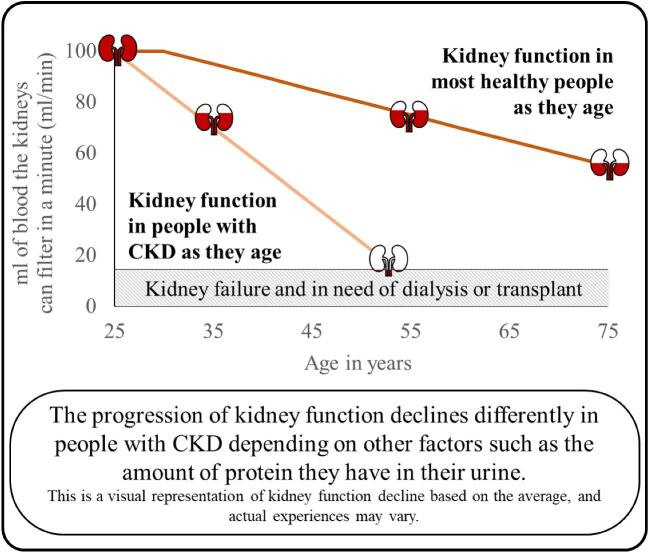
How does kidney function decline in healthy people versus people with CKD?

- Chronic kidney disease (CKD for short) is a progressive and permanent condition where kidneys slowly wear out over time and lose the ability to remove the body's waste products from the blood.
- In healthy people, the filtering abilities of the kidneys starts to slowly decline around the age of 30.
- In people with CKD, there is often a faster decline in kidney function which can eventually lead to kidney failure, where patients may need to start kidney replacement therapy such as dialysis treatment (a regular procedure to remove dangerous waste products from the blood) or a kidney transplant (Fig. 1).

### What causes CKD?

- CKD is caused by conditions that put a strain on the kidneys. Conditions which could cause CKD include (but are not limited to):
  ○ diabetes,
  ○ heart failure,
  ○ high blood pressure,
  ○ excess body weight,
  ○ kidney inflammation (also known as glomerulonephritis),
  ○ inherited conditions such as cysts in the kidneys, which can make the kidneys larger (polycystic kidney disease) and
  ○ blockage in the flow of urine, which may be caused by kidney stones or an enlarged prostate.

### What are the symptoms of CKD?

**Figure 2:**
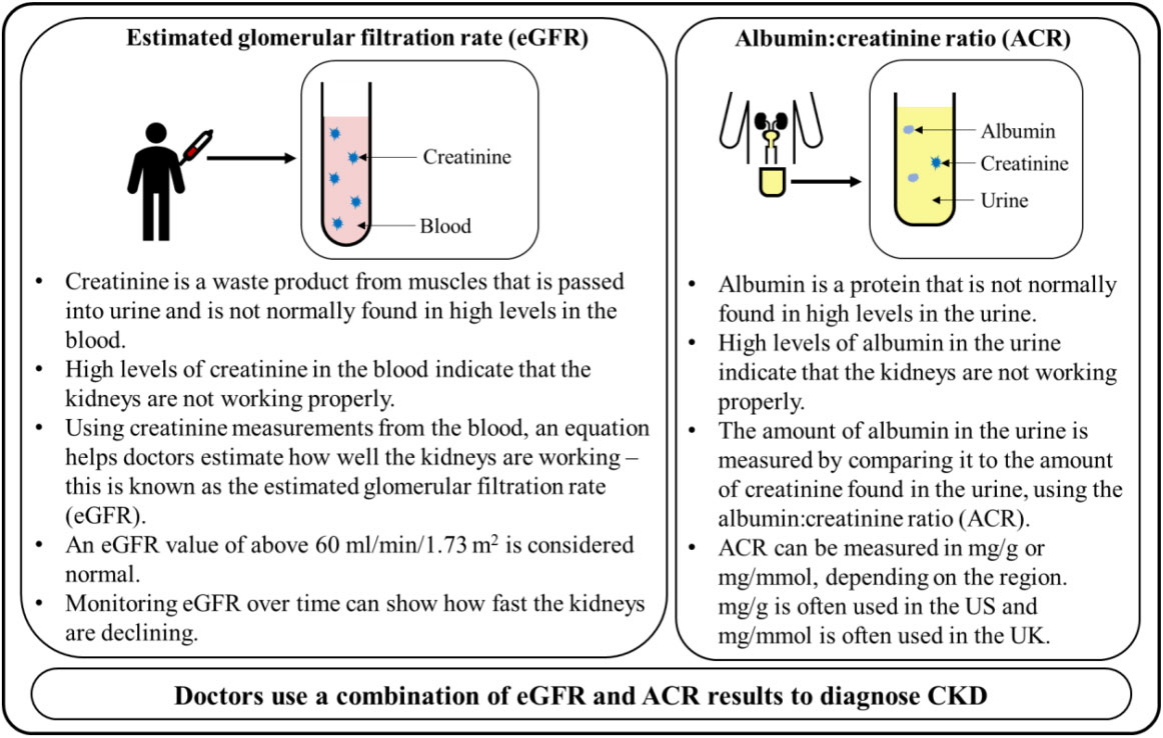
How do doctors diagnose CKD?

- CKD starts as a silent disease and has no specific symptoms in the early stages as the body can often cope with the loss of kidney function before symptoms develop. CKD is often diagnosed in the later stages (such as a loss of kidney function of more than 50%) from routine tests for other conditions that put people at higher risk of developing CKD (Fig. 2).
- In later stages of CKD, the following symptoms tend to develop:
  ○ poor appetite and weight loss,
  ○ feeling tired and having less energy,
  ○ itching of the skin,
  ○ reduced urine output,
  ○ swollen feet and ankles due to excess fluid (edema) and
  ○ shortness of breath, which may be due to fluid in the lung or anemia.

### How do you treat CKD?

- There is no cure for CKD, but the decline of kidney function in people with CKD may be slowed down by:
  ○ eating a healthy diet, moderate exercise and stopping smoking,
  ○ diagnosing and treating high blood pressure,
  ○ controlling blood sugar levels in diabetes,
  ○ avoiding medications that may harm the kidneys and
  ○ using certain medications that slow the decline of kidney function, such as renin–angiotensin system (RAS for short) inhibitors.
- People who have CKD are more likely to develop other conditions such as diseases affecting the heart or blood vessels (cardiovascular disease). The risk of cardiovascular disease increases as kidney function lessens, so it is important that the progression of CKD is slowed down, and patients are often offered statin-based therapy to help reduce the risk of heart attacks or strokes.
- Because there are few medications available that are effective at slowing kidney disease progression and improving the health or quality of life of patients living with CKD, it is important that doctors and researchers continue to look for effective treatments for the condition.

### *Patient perspective:* what is it like living with CKD?

Living with CKD has its challenges as life as you knew it changes. It's hard to explain to others how you feel; even though the disease doesn't give you any external symptoms, you could experience emotional and physical issues such as low mood and fatigue. However, CKD can be managed well by adjusting your diet, taking your medication regularly, and something that made a huge difference to me was educating myself about the disease and understanding my blood results.

Jose Rico-Diaz, patient contributor

### What is empagliflozin?

**Figure 3:**
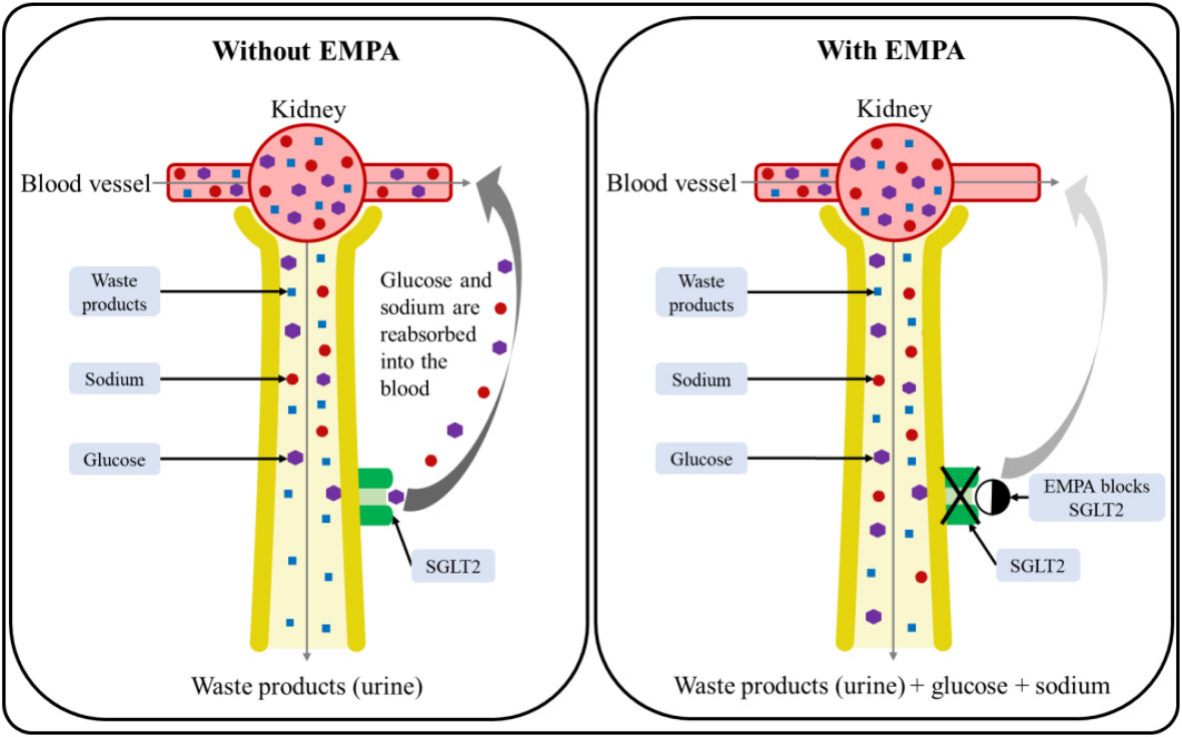
How does EMPA work?

- Empagliflozin (pronounced EM-pah-glih-FLOW-zin, shortened to EMPA) is a drug that was developed to reduce blood sugar levels in people with diabetes by increasing the amount of sugar passed into the urine.
  ○ EMPA does this by blocking a channel in the kidney called sodium-glucose co-transporter 2 (SGLT2), which normally reabsorbs sugar from the urine to prevent calorie loss (Fig. 3). EMPA is known as an SGLT2 inhibitor.
  ○ By increasing the amount of glucose removed by the kidneys, treatment with EMPA reduces the amount of glucose in the blood in people with type 2 diabetes.
- Although originally studied as a treatment for type 2 diabetes, several clinical trials have shown that EMPA also has a positive effect on heart and kidney function in people with and without diabetes.
- ○ EMPA allows salt (sodium) to be passed into urine with glucose. Researchers think that this reduces blood pressure.
- ○ The EMPA-KIDNEY trial was the largest trial of this type of drug to investigate effects specifically on kidney disease progression in people with CKD.

## METHODS

### What is the EMPA-KIDNEY trial?

- The EMPA-KIDNEY trial was designed to see whether EMPA could protect the kidneys and heart in a broad range of different types of people with CKD, including people with and without diabetes, people with CKD of different causes, and people with a wide range of estimated glomerular filtration rate (eGFR) and albumin to creatinine ratio (ACR).
  ○ People with CKD could participate if they had:
    ▪ eGFR of 45 to 90 mL/min/1.73 m^2^ in combination with a ACR 200 mg/g (or 22.6 mg/mmol) or more, or
    ▪ eGFR of 20 to 45 mL/min/1.73 m^2^ regardless of the level of ACR.
  ○ People with polycystic kidney disease or who had previously received a kidney transplant were unable to participate in the EMPA-KIDNEY trial.
- Participants were randomly (like tossing a coin) split into two equal groups. The groups received either 10 mg of EMPA or a pill that looked like EMPA but contained no medicine (called a placebo) every day, in addition to their normal therapies.
  ○ During the study, neither the participants nor the researchers knew who was taking the EMPA pill or who was taking the placebo. (This is called a double-blind trial.)
- When analyzing the trial, the international research teams planned to look at:
  ○ either kidney disease progression or dying from cardiovascular disease; kidney disease progression was defined as:
    ▪ those who started dialysis or received a kidney transplant during the trial,
    ▪ those who died during the trial because of kidney failure, or
    ▪ those who had a specific change in eGFR during the trial:
    • decline in eGFR to less than 10 mL/min/1.73 m^2^,
    • decline in eGFR of 40% or more from the value at the start of the trial
  ○ how quickly eGFR declined,
  ○ the number of participants who were hospitalized regardless of the cause,
  ○ the number of participants who died regardless of the cause,
  ○ the number of participants who experienced cardiovascular disease or died (hospitalized for heart failure or died from cardiovascular disease) and
  ○ the number of side effects.
- After the study was completed, the researchers found out who was taking EMPA or placebo and compared the results to see what the effect of treatment with EMPA was in people with CKD.
- As well as comparing the effect of EMPA overall, researchers also studied the effects of EMPA in specific groups of participants. The main groups that were looked at in more detail were:
  ○ people with and without diabetes,
  ○ people with different levels of eGFR at the start of the trial and
  ○ people with different levels of ACR at the start of the trial.
- The EMPA-KIDNEY trial ran from 2019 to 2022.

## RESULTS [1]

**Figure 4:**
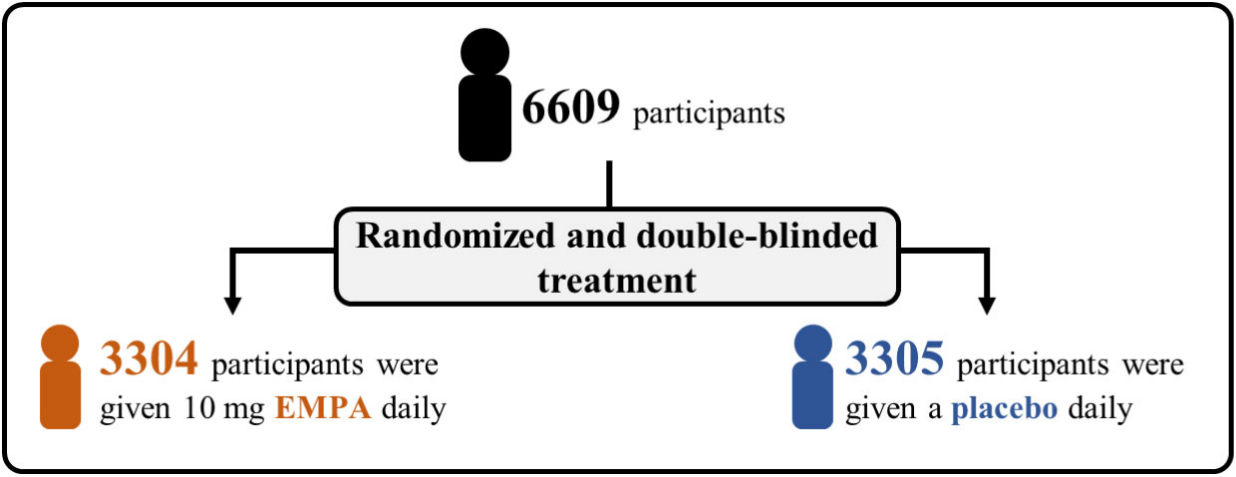
Who took part in the study?

**Figure 5:**
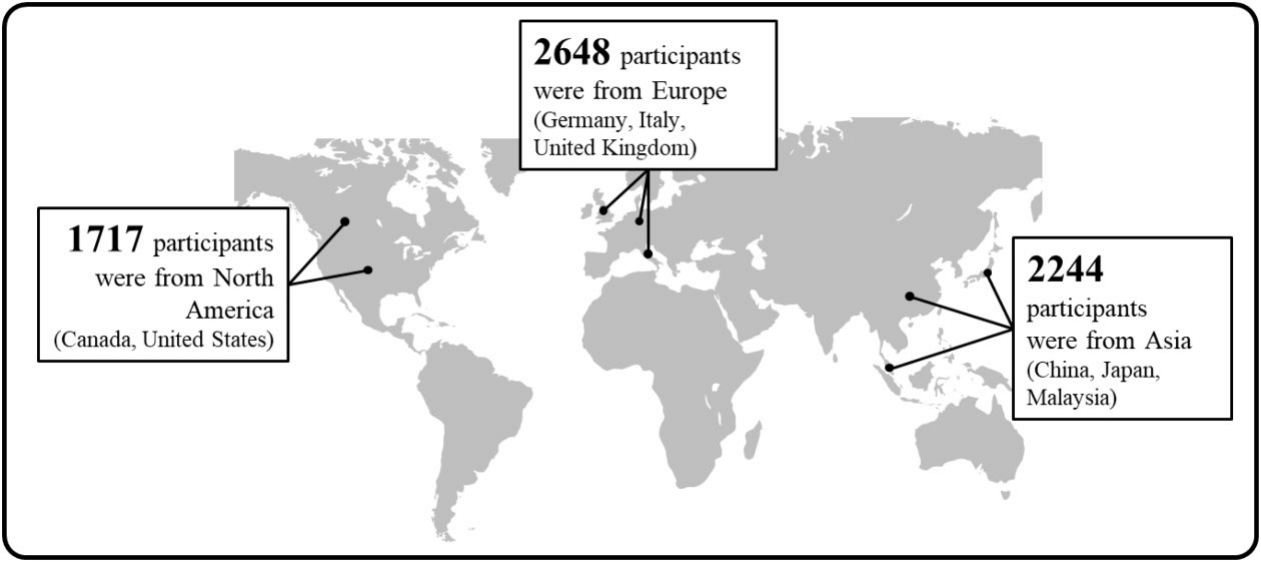
Where were the participants from?

**Figure 6:**
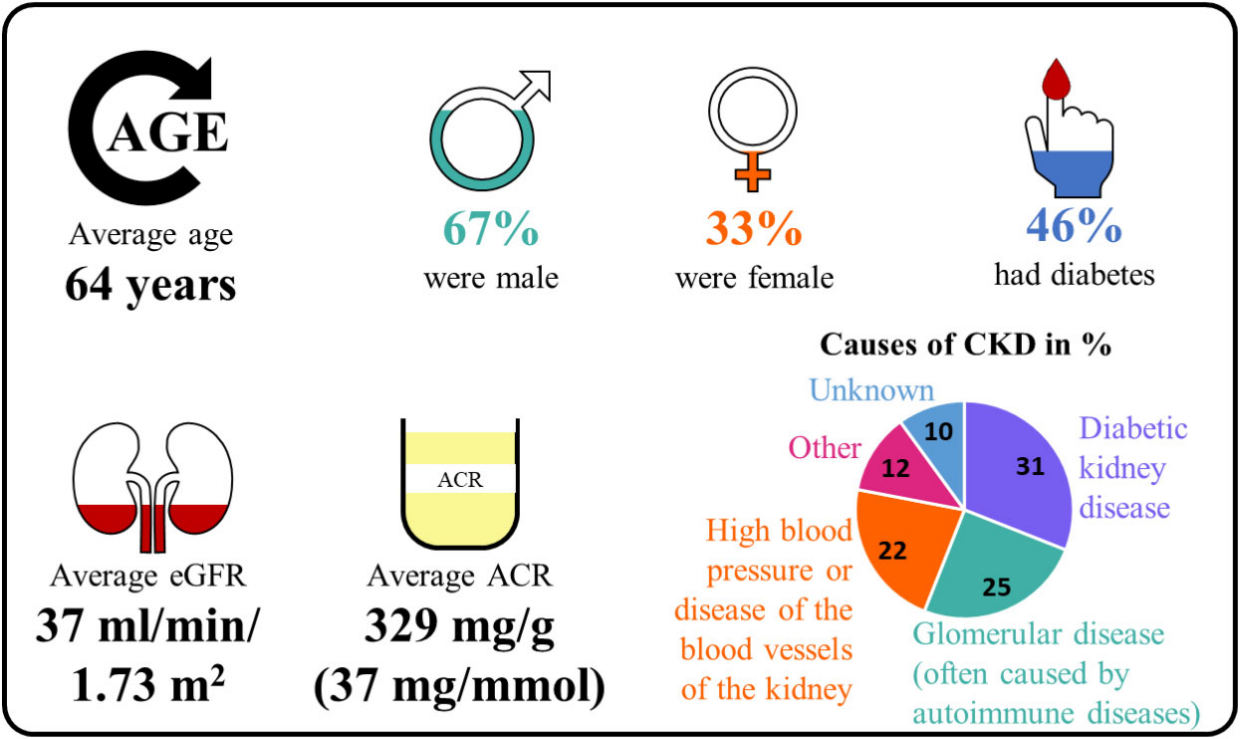
What were the characteristics of the participants at the start of the EMPA-KIDNEY trial?

**Figure 7:**
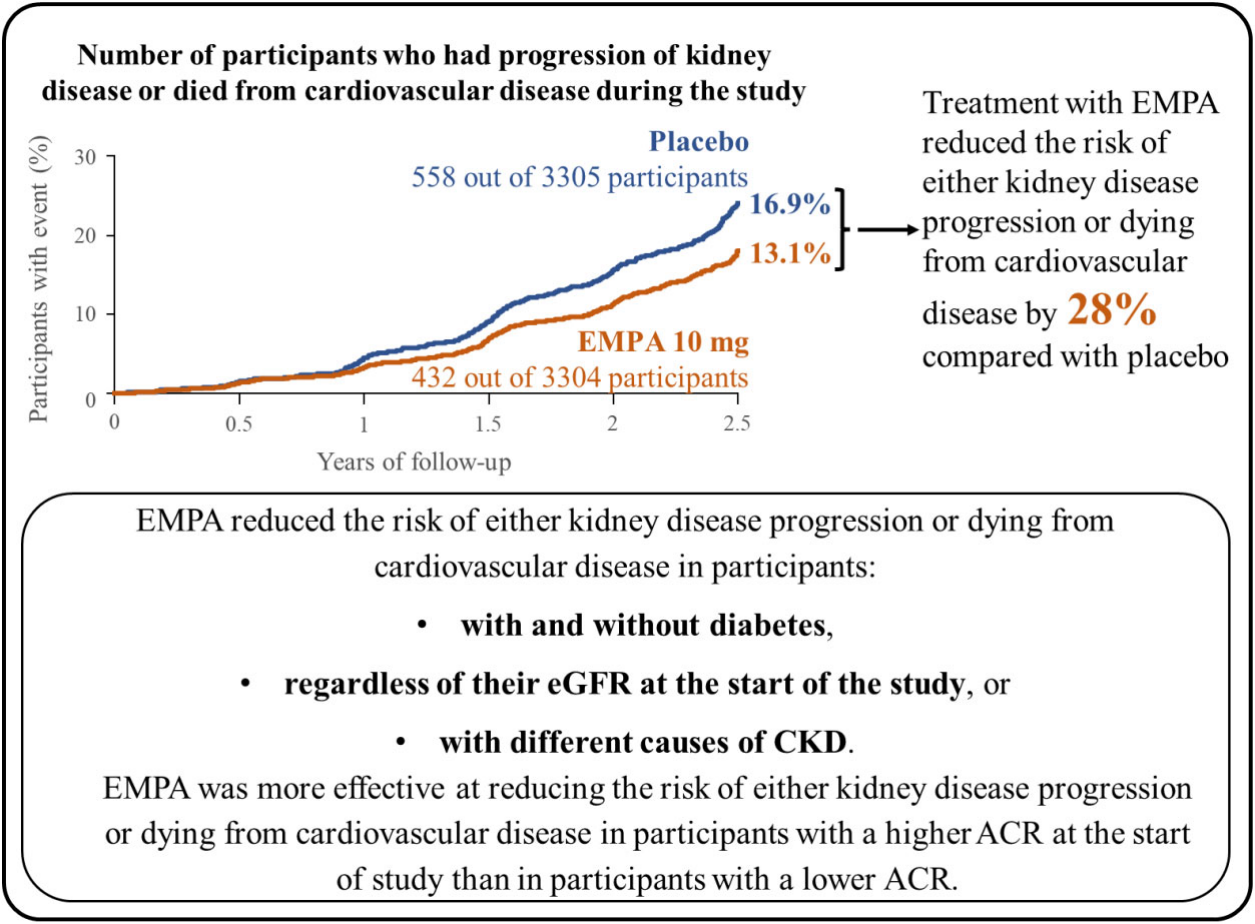
How did treatment with EMPA affect kidney disease progression or dying from cardiovascular disease? The reduced risk of either kidney disease progression or dying from cardiovascular disease result was tested using a statistical test and showed that there was a less than 1 in 1000 probability that the results was due to chance.

**Figure 8:**
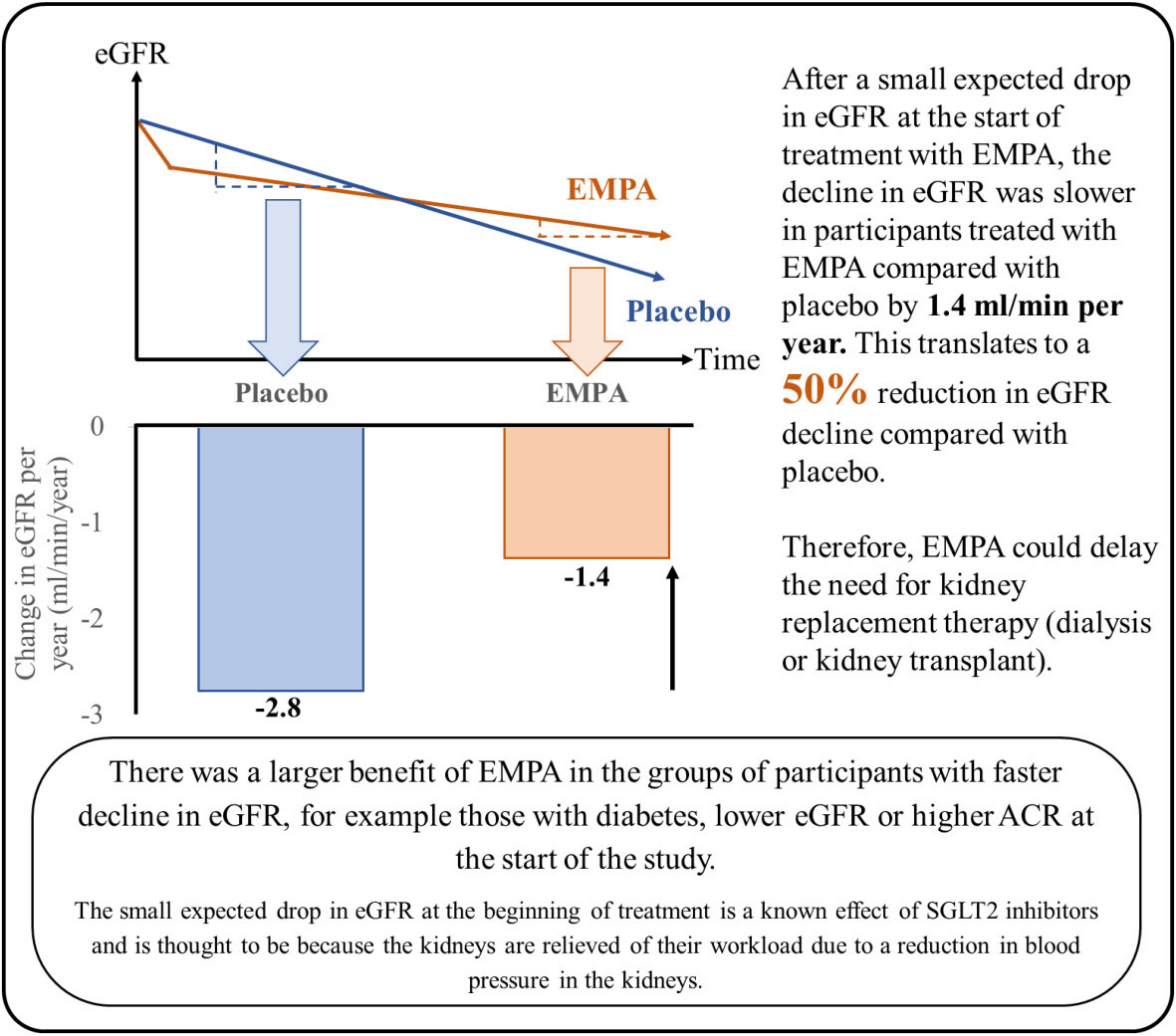
How did treatment with EMPA affect eGFR decline?

**Figure 9:**
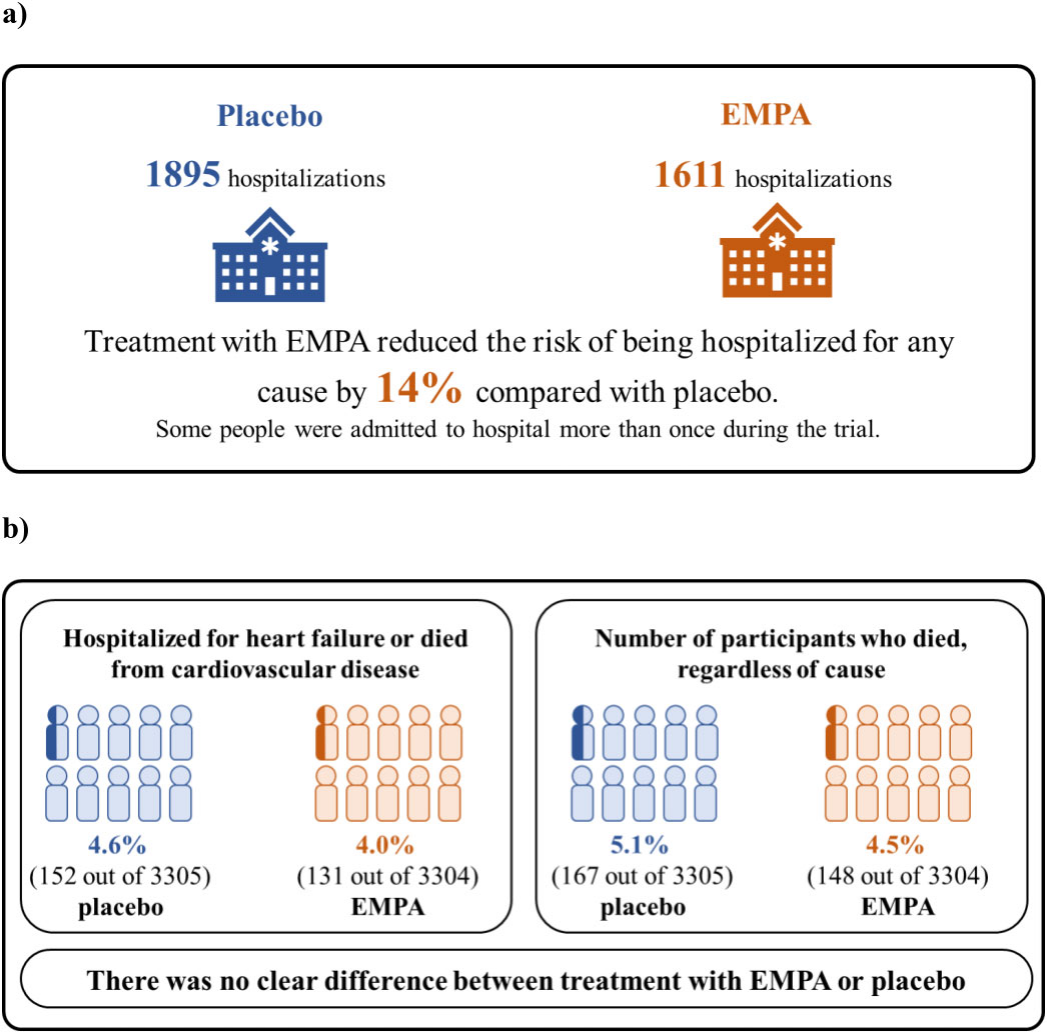
How did treatment with EMPA affect the number of times participants were hospitalized for any reason (**a**), hospitalized for heart failure or died from cardiovascular disease, or the numbers who died regardless of the cause (**b**)?

**Figure 10:**
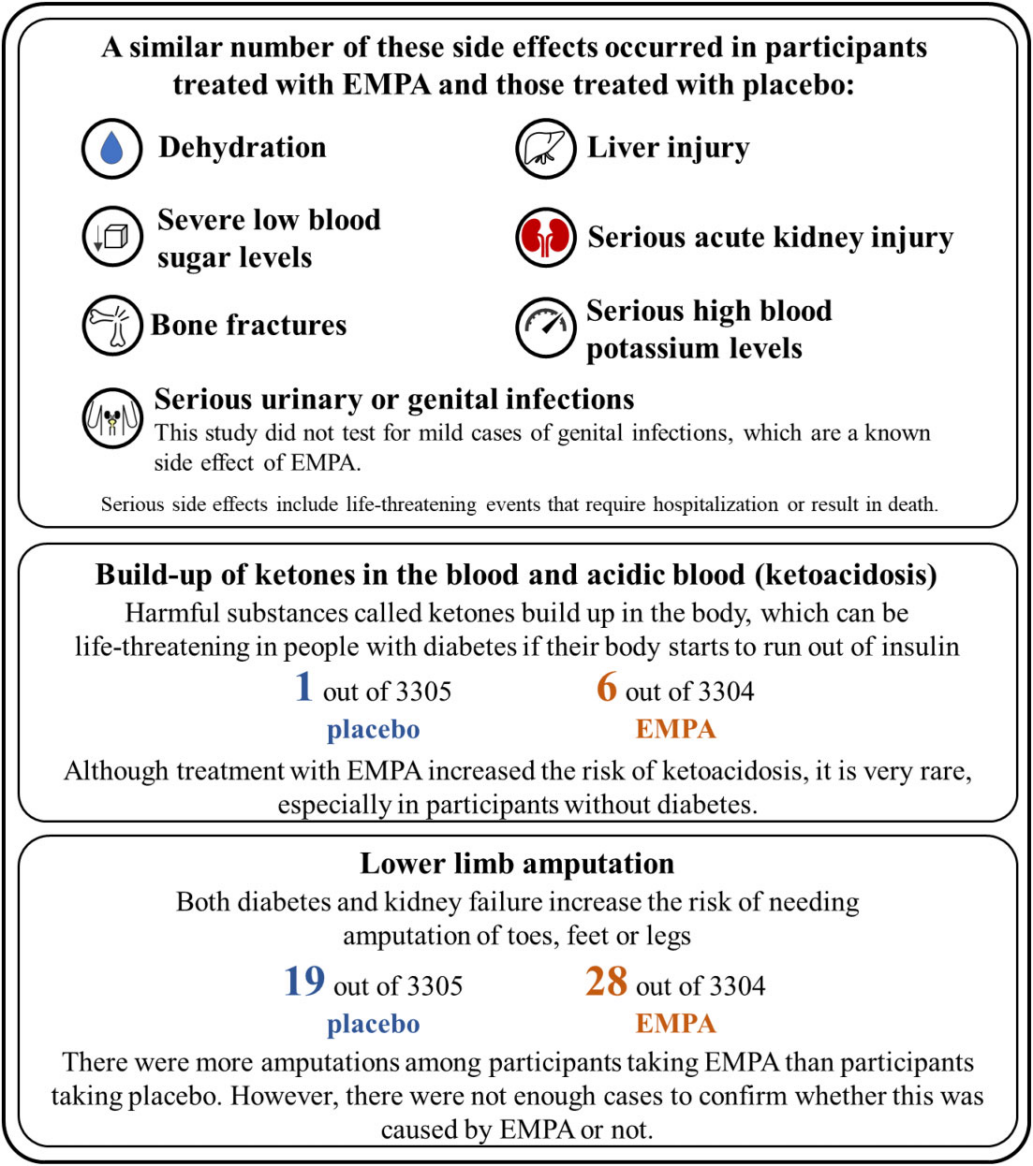
How safe is EMPA?

- There were 6609 participants at the start of the trial: 3304 participants were given 10 mg EMPA daily and 3305 participants were given a placebo daily (Fig. 4).
- Participants were recruited from different parts of the world, including North America, Asia and Europe (Fig. 5).
- At the start of the EMPA-KIDNEY trial, 46% of participants had diabetes, the average eGFR was 37 mL/min/1.73 m^2^, and the average ACR was 329 mg/g (or 37 mg/mmol) (Fig. 6).
- By the end of the trial, 558 out of 3305 participants in the placebo group (16.9%) and 432 out of 3304 participants in the EMPA group (13.1%) had progression of their kidney disease or died from cardiovascular disease. Overall, treatment with EMPA reduced the risk of either kidney disease progression or dying from cardiovascular disease by 28% compared with placebo (Fig. 7).
- Treatment with EMPA reduced the rate of eGFR decline by 50% per year compared with placebo (Fig. 8).
- Treatment with EMPA reduced the risk of being hospitalized for any cause by 14% compared with placebo (Fig. 9a).
- This study did not show a difference between treatment with EMPA or placebo in:
  ○ being hospitalized for heart failure or dying from cardiovascular disease) or
  ○ the number of participants who died, regardless of the cause (Fig. 9b).
    ▪ This may be because such events were uncommon in the trial, making it difficult to measure differences between the groups.
- EMPA was well-tolerated: 1 year into the trial, 9 of every 10 participants allocated to EMPA (or placebo) were still taking their study treatment regularly.
- A similar number of the side effects studied in the trial occurred in participants taking EMPA and participants taking placebo (Fig. 10).

## CONCLUSIONS

- Participants who received EMPA were less likely to have progression of their kidney disease or die from cardiovascular disease versus those receiving placebo.
  ○ This reduction was shown in a range of participants, including those with or without diabetes, those with different levels of eGFR and those with different causes of kidney disease.
- The benefits of EMPA clearly outweighed the side effects and serious side effects such as ketoacidosis were extremely rare.
- The results show that treatment with EMPA is effective and is safe in a broad range of people, including those with lower levels of kidney function.

### *Patient perspective:* what do the results mean to me?

A medicine that helps prevent me from suffering from kidney disease would be very welcome. I know the risks that I run as a person with diabetes and know there is little I can do to avoid the disease if it should attack me. Knowing that there is a fairly high chance of being helped with this medicine is quite a consolation. The research could help many people who are living with diabetes, and I welcome its introduction.

Dianna Moylan, patient contributor

## Data Availability

Data will be made available to bona fide researchers in line with the policy and procedures described at: https://www.ndph.ox.ac.uk/data-access. In adherence with the Boehringer Ingelheim Policy on Transparency and Publication of Clinical Study Data, scientific and medical researchers can request access to clinical study data, typically, one year after the approval has been granted by major regulatory authorities or after termination of the development programme. Researchers should use the https://vivli.org/ link to request access to study data and visit https://www.mystudywindow.com/msw/datasharing for further information.

## References

[1] The EMPA-KIDNEY Collaborative Group; Herrington WG, Staplin N, Wanner C et al. Empagliflozin in patients with chronic kidney disease. N Engl J Med 2023;388:117–27. 36331190 10.1056/NEJMoa2204233 PMC7614055

